# Amyloid-β and Astrocytes Interplay in Amyloid-β Related Disorders

**DOI:** 10.3390/ijms17030338

**Published:** 2016-03-04

**Authors:** Yazan S. Batarseh, Quoc-Viet Duong, Youssef M. Mousa, Sweilem B. Al Rihani, Khaled Elfakhri, Amal Kaddoumi

**Affiliations:** Department of Basic Pharmaceutical Sciences, School of Pharmacy, University of Louisiana at Monroe, Monroe, LA 70504, USA; batarsy@warhawks.ulm.edu (Y.S.B.); duongqa@warhawks.ulm.edu (Q.-V.D.); mousaym@warhawks.ulm.edu (Y.M.M.); alrihasb@warhawks.ulm.edu (S.B.A.R.); hamadgh@warhawks.ulm.edu (K.E.)

**Keywords:** Alzheimer’s disease, cerebral amyloid angiopathy, Down syndrome, frontotemporal dementia, astrocytes, Amyloid-β

## Abstract

Amyloid-β (Aβ) pathology is known to promote chronic inflammatory responses in the brain. It was thought previously that Aβ is only associated with Alzheimer’s disease and Down syndrome. However, studies have shown its involvement in many other neurological disorders. The role of astrocytes in handling the excess levels of Aβ has been highlighted in the literature. Astrocytes have a distinctive function in both neuronal support and protection, thus its involvement in Aβ pathological process may tip the balance toward chronic inflammation and neuronal death. In this review we describe the involvement of astrocytes in Aβ related disorders including Alzheimer’s disease, Down syndrome, cerebral amyloid angiopathy, and frontotemporal dementia.

## 1. Introduction

Amyloid-β (Aβ) peptide was primarily characterized as part of the pathological hallmark of Alzheimer’s disease (AD) and Down syndrome (DS) [1,2]. Recently, Aβ has been recognized in other neurological disorders. The origin of Aβ peptide comes from a membrane bound protein called amyloid precursor protein (APP) which after sequential cleavage by secretase-β and secretase-γ produces extracellular Aβ. Secretase-γ cleavage could occur in various locations producing different forms of Aβ containing different numbers of amino acids, with the major form being Aβ_40_ followed by Aβ_42_. The latter is more hydrophobic with a higher propensity of creating amyloid oligomers and insoluble fibrils [3].

Astrocytes are glial cells known for their star-like appearance and play a key role in neurological diseases. Astrocytes have a critical role in providing the needed requirements for neurons to function properly. They have a distinctive anatomical location covering the endothelial cells of the blood-brain barrier (BBB), and form a network of fine lamellae closely opposed to the outer surface of the endothelium thus creating the perivascular space [4,5]. As a result of their critical location, astrocytes are involved in the transport of proteins, glucose, and toxic byproducts across the BBB [6,7]. In addition, astrocytes secrete signaling molecules involved in the expression of proteins important to the function and intactness of the BBB such as the sonic hedgehog, retinoic acid, glial derived neurotrophic factor (GDNF) and angiopoietin 1 [6,7]. These factors enhance the BBB intactness via the activation of multiple receptors expressed in the endothelial cells, which lead to increased expression of tight junction proteins manifested by higher transepithelial electrical resistance (TEER) values [8,9,10,11,12]. Astrocytes are important cellular component of the neurovascular unit, working hand in hand with the endothelial cells, neurons, microglia, and pericytes to maintain a state of tight regulation sensitive enough to detect neuronal metabolic and energy changes [13]. Neurovascular coupling and vascular tone are maintained by an astrocytic Ca^2+^ rise combined with phospholipase A2 activation that lead to the release of arachidonic acid, later converted to vasoactive signals such as prostaglandins and epoxyeicosatrienoic acids [14,15]. Localized conditions such as arteriolic tone can determine the function of these released signaling molecules to either work as vasoconstrictors or dilators. Astrocytes are also involved in the synaptic area where neurons meet for signal transmission to sense metabolic requirement changes associated with synaptic activity [16]. Astrocytes have an important role in energy regulation where they express the glucose transporter (GLUT1) to uptake glucose and convert it to lactate utilized by the neurons as an energy source [17]. Lactate regulation is maintained by astrocytes’ glutamate transporters which co-uptake synaptic glutamate and Na^+^ ions (one ion for each transported glutamate). This will result in more Na^+^ load that activate the Na^+^/K^+^ ATPase pump to re-stabilize normal Na^+^/K^+^ balance causing a state of temporary ATP depletion, which consequently induces an increase in glucose uptake and lactate generation [18]. Moreover, astrocytes are responsible for the rapid clearance of glutamate via the expression of its transporter glutamate transporter-1 (GLT-1) [19]. The efficiency in glutamate removal from the synaptic area is essential to prevent its overexposure as glutamate excitotoxicity has been involved in different pathological events including electrolytes imbalance and apoptosis [20,21].

In this review we will focus on the interaction between Aβ and astrocytes in Aβ related pathological disorders, including Alzheimer’s disease (AD), cerebral amyloid angiopathy (CAA), Down syndrome (DS) and frontotemporal dementia (FTD). While these diseases differ in their clinical manifestations, all are associated with Aβ deposition. Thus, while greater evidence is available in the literature for AD, the astrocyte-Aβ interaction is expected to be applicable to CAA, DS and FTD as it is with AD.

## 2. Amyloid-β Related Disorders

### 2.1. Alzheimer’s Disease

Alzheimer’s disease (AD) is considered one of the most common neurodegenerative disorders affecting the elderly population. With increasing life expectancy, the number of AD patients is expected to increase significantly over the coming decades. Currently, in the United States, AD affects five million individuals, and it is expected to rise to 16 million by 2050 [22]. Unlike other major high morbidity and mortality disorders such as cancer and cardiovascular disease, AD patients have very limited therapeutic options that include the acetylcholinesterase inhibitors (ChEIs) rivastigmine, donepezil, and galantamine in addition to the *N*-methyl-d-aspartate (NMDA) receptor antagonist memantine [23]. AD pharmacological treatment is limited due to lack of effective drugs to slow down the progression of or treat the disease to improve patient’s quality of life [24]. 

AD is clinically characterized by memory loss and learning abnormalities [25]. Histopathological analysis of the brains of affected AD patient’s showed extracellular insoluble deposits of Aβ peptides in addition to intracellular accumulation of neurofibrillary tangles composed of hyperphosphorylated tau aggregates [26,27]. In addition, AD patients show significant synaptic loss and neuronal death starting in the hippocampus and entorhinal cortex and spreading into different areas in the brain parenchyma [28]. Abnormal Aβ accumulation due to over production in familial AD [29], or impaired clearance in sporadic AD [30], is proposed to initiate a cascade of events including the formation of Aβ oligomers and fibrils with the latter being identified as the major component of Aβ plaques [26]. Due to its hydrophobicity and propensity to aggregate, amyloid oligomers and insoluble fibrils are mostly formed from Aβ_42_ [3]. Thus, in AD patients CSF levels of Aβ_42_ are lower than those of cognitively normal subjects due to its increased brain deposition and diminished number of neurons producing Aβ_42_ [31,32].

Two main pools of Aβ have been distinguished in the brain of AD patients, a soluble pool that consists of a mixture of Aβ monomers and soluble oligomers, and an insoluble pool of insoluble oligomers and higher order histologically prominent insoluble Aβ fibrils. Increasing evidence indicates that soluble Aβ is biologically more active than the insoluble Aβ fibrils. Moreover, a comparison of pathology with clinical diagnosis of AD brains found a weak correlation between Aβ plaque load and the progression of AD symptoms, in contrast to a better correlation of the soluble pool of Aβ with AD clinical severity [33,34]. The pathological accumulation of Aβ in the brain parenchyma of AD patients is associated with a strong inflammatory process [35,36]. Higher molecular weight Aβ aggregate forms have been associated with many inflammatory pathways and glial cells activation [37]. The interaction between astrocytes and Aβ is a complex process where different aggregation forms of Aβ react with astrocytes differently [38]. Several studies in the literature have shown the co-localization of inflammation promoting astrocytes (*i.e.*, activated astrocytes) with insoluble Aβ plaques in the brain parenchyma of AD patients [39,40,41]. Before the formation of insoluble Aβ plaques, Aβ forms an intermediate species in the oligomeric configuration [26]. The randomness and abundance of Aβ oligomers give rise to the possibility that some of those formed Aβ oligomers resemble an endogenous ligand and interfere with the function of its receptors. This process initiates several pathological pathways including tau hyperphosphorylation and down-regulation of several proteins involved in cell homeostasis; the Aβ complexes causing this phenomenon are called amyloid derived diffused ligands (ADDLs) [42,43,44]. ADDLs affect the function of astrocytes in regulating synaptic glutamate levels by down-regulating GLT-1 expressed on the astrocytic ends involved in the synaptic area [45]. Moreover, Aβ oligomers and fibrils have the ability to reduce the release of glutathione, the major antioxidant in the CNS, from astrocytes indicating that Aβ forms other than monomers may change brain oxidative balance by interfering with glutathione release [46].

Astrocytes mediate the clearance of Aβ by multiple mechanisms [38,47,48,49,50,51,52,53,54,55,56,57]. Compared to Aβ oligomers and monomers, fibril uptake by astrocytes is limited [38]. A comparison study between Aβ oligomers uptake vs fibrils demonstrated that oligomers have a significantly higher uptake while fibrils showed less cell membrane actin filaments involvement, suggesting their adherence on the surface rather than being taken up [38]. Astrocytes express high number of transport proteins and receptors that are capable of the uptake of Aβ monomers such as low density lipoprotein receptor-related protein (LRP1), scavenger receptor class B member 1 (SCARB1), and Receptor for Advanced Glycation End Products (RAGE) [48,49,50]; however, not all Aβ monomer transport proteins are capable of oligomers uptake. LRP1, for example, is effective in Aβ monomers uptake but not Aβ oligomers [48], while SCARB1 interacts with fibrillar Aβ [49]. RAGE, on the other hand, is capable of interacting with the three forms of Aβ [51]. Beside transport proteins, astrocytes mediate the clearance of Aβ directly by endocomal-lysosomal pathways subjecting Aβ to degradation by a variety of degrading enzymes such as insulin-degrading enzyme (IDE) and neprilysin (NEP) that are only effective on the degradation of Aβ monomers [52,53], while matrix metalloproteinase-9 (MMP9) is effective against both monomers and fibrils [54]. Astrocytes also contribute to the clearance of parenchymal Aβ either directly by releasing degrading enzymes extracellularly [47], or indirectly by secreting ApoE which acts as a chaperone protein to facilitate the clearance of Aβ via LRP1 expressed on the astrocytes themselves [48,55], or on brain endothelial cells facilitating Aβ clearance across the BBB [56]. Another reported astrocytic clearance pathway of Aβ is by phagocytosis via the PEA15 (phosphoprotein enriched in astrocytes 15 kDa), a receptor that is upregulated by high levels of brain Aβ [57]. Microglia can clear Aβ fibrils by phagocytosis [58], which is attenuated by Aβ oligomers suggesting that Aβ oligomers can further support and promote Aβ accumulation by inhibiting microglial clearance. Additionally, microglia can clear soluble Aβ by micropinocytosis via multiple receptors such as P2Y4 [59].

Collectively, astrocytes evidently play an important role in reducing Aβ brain parenchymal load to maintain normal levels, however, when accumulated Aβ plaques reach a certain threshold, which could result in the death of astrocytes surrounding the plaques and formation of astrocyte-derived amyloid plaques or glial fibrillary acidic protein (GFAP) positive plaques [60,61]. The area stained positively with GFAP correlates with the presence and involvement of activated astrocytes [62]. GFAP is an intermediate filament protein, and its immunostaining is largely used as a marker for astrocytes, and to co-localize astrocytes with Aβ plaques [63,64]. In response to inflammation GFAP intensity increases as a result of astrocytes activation and remodeling into their traditional activated star-like shape with thick and extended branches [63,64]. While the mechanism behind GFAP up-regulation in response to astrocytes activation is not fully understood, available studies suggested that upon activation, astrocyte production of inducible nitric oxide synthase (iNOS) increases which in turn increases the release of nitric oxide (NO) responsible for GFAP increased levels [65,66]. Although GFAP is the most widely used marker for astrocytes, it has some limitations as not all astrocytes are GFAP positive [67], thus other astrocytic markers such as glutamate aspartate transporter (GLAST) and aldehyde dehydrogenase 1 family member L1 (Aldhl1) could alternatively be used [68,69]. Astrocyte activation induces intracellular electrolyte changes that involve calcium wave formation [70,71,72]. *In-vitro* evidence suggests that familial AD mutation alters the sensitivity of astrocytes toward the formation of calcium waves in response to ATP and glutamate [73], indicating that AD-astrocytes are more sensitive than normal astrocytes in response to an activating factor. Thus, Aβ involved astrocytes may become more of inflammatory cells and neglect their neuro-supportive role [56,65,74,75].

The role of Aβ induced reactive astrogliosis has been documented in different studies; however, whether AD is caused by or partially influenced by reactive astrogliosis requires further investigations. Findings from *in vitro* studies tested the interaction of Aβ and astrocytes demonstrated the modulatory effect of Aβ on the expression of multiple astrocytic proteins associated with AD pathology such as APP and GLT-1 transporter. Aβ could increase its own production as a result of APP up-regulation [76], and was shown to reduce the expression of GLT-1 transporter and enhance glutamate synaptic toxicity [45]. These results suggest that Aβ-astrocyte interaction poses a pathological risk and contributes to AD. Furthermore, additional studies indicated a possible link of increased Aβ levels and astrocytes activation with subsequent increase in reactive nitrogen and oxygen species (RNS, ROS), and induction of intracellular Ca^2+^ levels which could further increase ROS [65,77]. Besides, in astrocyte-neuron co-culture, Aβ-induced astrocytes activation increased the release of inflammatory mediators (such as IL-1β, IL-6, and TNF-α) from astrocytes that caused neurotoxicity suggesting a close association between cytokine release and the neurotoxic events that occur downstream of Aβ [78]. The increased number and/or reactive shape of astrocytes located in close proximity to Aβ plaques, thus, could indicate astrocyte involvement in local inflammation rather than for direct uptake and degradation of Aβ [63,64]. Yet, whether astrocytes actually migrate to the site of Aβ plaques or it is just a matter of altered morphology of already existing astrocytes around the plaques remains a debatable matter. Recent evidence supports the latter, where the authors demonstrated that astrocyte distribution in the brains of an AD mouse model with Aβ plaques was not different from that in wild type mice [79]. Collectively, the chronic activation of astrocytes in response to Aβ associated inflammation is harmful, thus reducing their activation is a novel therapeutic approach to restore their supportive functions and prevent further inflammation-mediated cell death.

### 2.2. Cerebral Amyloid Angiopathy

Cerebral amyloid angiopathy (CAA) is characterized by Aβ deposition along the walls of the cerebral vasculature, which includes arteries, arterioles, veins and less often capillaries [80]. CAA is a frequent pathological anomaly and a fairly common clinical entity in the elderly. As a detectable pathology, cerebrovascular amyloid is present in approximately 10% to 40% of elderly brains and 80% or more in brains of AD patients [81]. CAA is caused by a several biochemical and genetic CNS disorders such as mutations in the APP or presenilin genes [3]. The amphiphilic nature of Aβ prevents its transport across the BBB unless mediated by specialized carriers and/or receptor transport proteins like LRP1 [82,83], RAGE [84], and P-glycoprotein (P-gp) [85,86] that are expressed on the surface of the brain capillary of endothelial cells and contribute to the transport of Aβ [82,83,84,85,86]. These mechanisms control the brain levels of Aβ and regulate its clearance [87]. The imbalance between Aβ production and clearance results in increased levels of brain Aβ and deposition, which can lead to a cascade of events including, among others, astrocytes activation, release of inflammatory components, activation of the complement systems, oxidative stress, alteration of the BBB permeability, and neural toxicity [88,89]. In CAA, non-fibrillar monomeric and oligomeric Aβ were demonstrated to deposit around the smooth muscle cells along the blood vessels [90]. Amyloid deposition can weaken cerebral blood vessels walls, causing rupture and therefore leading to both asymptomatic microbleeds and lobar intracerebral hemorrhage (ICH) [87]. Furthermore, Aβ deposition can obliterate the vessel lumen, leading to ischemia and related clinical manifestations [91]. Focal neurological deficits, disturbances of consciousness, progressive cognitive decline, dementia, and death can occur as a consequence of these vascular mechanisms [91,92].

The end-feet of astrocytes are in close association with the endothelial cells, and form a lacework of fine lamellae closely associated to the outer surface of the endothelium [5]. This close anatomical association indicates a role of astrocytes in development of the specialized BBB phenotype of the brain endothelium [93]. The BBB phenotype includes complex tight junctions and specific transport and enzyme systems which regulate molecular traffic across the endothelial cells [94]. Astrocytes also play a key role in neurovascular diseases associated with Aβ such as CAA. In CAA, in response to aggregated Aβ around the neurovascular unit, reactive astrocytes will activate transcription factor nuclear factor-kappa B (NF-κB) and increase the expression of TNF-α, IL-1β, and cyclooxygenase-2 (COX-2) and other inflammatory cytokines [95]. The interaction of endothelial cells with these inflammatory cytokines could result in a neuroinflammatory response that alters the expression of tight junction proteins and disrupts the BBB integrity, which is one of the characteristics of CAA [96]. Reactive astrocytes have also been shown to increase the expression of Aβ degrading enzymes, such as secretase-β (BACE-1), NEP, IDE and angiotensin-converting enzyme (ACE), all necessary to lighten the burden of Aβ [97]. Another group of Aβ degrading enzymes known as matrix metalloproteinases (MMPs) were also investigated in an aged APP/presenilin mouse model. The results of this study confirmed the hypothesis that activated astrocytes do express and secrete MMPs, in particular MMP-2 and MMP-9, and that MMPs do play a role in astrocyte-mediated Aβ degradation [98]. However, MMPs disrupt the tight junction proteins that create the basement membrane for the endothelial vasculature thus contributing to the opening of the BBB and the inflammatory process [99]. In addition, vascular oxidative stress from activated astrocytes may contribute to the impairment of the BBB and play a role in CAA. Evidence supports that ROS such as NADPH oxidase-derived ROS plays a key role in CAA induced cerebral vasculature dysfunction such as reduced baseline cerebral blood flow (CBF) and decreased CBF responses to topical vasodilators [100,101]. Collectively, Aβ-astrocytes interaction induces inflammatory cytokines, metabolizing enzymes, and reactive oxygen species that in turn further contribute to the neuroinflammation observed in CAA [95,96,97,98,99,100,101]

### 2.3. Down Syndrome

Down syndrome (DS) or trisomy 21, is the most well-known chromosomal defect disorder. According to the National Down Syndrome Society, one in every 691 babies in the United States is born with DS. The overexpression of APP gene located on chromosome 21 leads to early onset Aβ plaques in DS. Despite the fact that AD and DS are totally distinct disorders, the neuropathology is similar [102,103]. Thus, studies of DS patients could provide a unique opportunity to investigate the pathophysiological processes underlying the development of AD. Like AD, the pathophysiological hallmarks in DS include Aβ accumulation, neurofibrillary tangles, cerebrovascular pathology, white matter pathology, oxidative damage, and neuroinflammation [104]. Unlike AD, however, Aβ accumulates evenly in a wide spread distribution both in the entorhinal cortex and hippocampus of the DS brains [105], while in AD, the pathology starts in the entorhinal cortex and then spreads into the hippocampus [106]. Moreover, DS patients who carry the ApoE ε4 genotype show more than double Aβ accumulation compared to non-ApoE ε4 patients [105].

Aβ plaques that are a common finding in adults with DS have also been noted in some children with DS [107,108]. Several clinical trials found that by the age of 40 almost all individuals with DS have sufficient Aβ plaques for an AD diagnosis [109,110]. Activated astrocytes overexpressing neuroinflammatory cytokines such as IL-1 and S100B have been seen in DS before AD-like neuronal and extracellular changes [102]. According to the presence of early and sustained overexpression of IL-1 in DS, as well as the role of IL-1 in regulating the expression of APP [111], it was suggested that the neuroinflammatory response is the key cause for the development of AD in individuals with DS [102,112]. Besides, due to the duplication of chromosome 21 genes, expression of S100B at 1.5-fold higher than normal levels would be expected in DS but not necessarily in AD [113]. S100B protein is an astrocyte-derived cytokine encoded by a chromosome 21 gene located in DS critical region. S100B is overexpressed in the brains of DS fetuses [102], and this overexpression continues throughout life [114]. S100B acts as a neurite extension factor, which suggests its important function in the normal growth and maintenance of neurons [110]. Royston and colleagues showed that the number of activated astrocytes overexpressing S100B showed a significant correlation with the numeric density of Aβ plaques [115], these findings together with the established functions of S100B, support the idea that S100B overexpression promotes amyloid plaque formation and progression in DS [115,116,117,118]. Increased levels of S100B were associated with increased production of APP [119]. Like Aβ, increased levels of APP could induce neuronal stress and neuroinflammation as a result of the elevated release of sAPPα, which activates glial cells and induces IL-1β expression and promotes astrogliosis that in turn further induces the expression of APP gene [116,119]. S100B protects neurons from insults and promotes survival following injury [120], and its elevated blood levels serve as a marker indicative of stroke severity, survival, and progression from hemorrhage to acute thrombosis as well as severity of traumatic brain injury [120]. Blood levels of S100B were reported to increase by minor head trauma in children and young adults [120]. In addition to S100B increased expression in activated astrocytes, observed in neurological diseases, astrocyte-derived S100B expression were also reported to significantly increase in systemic diseases such as mild to severe liver diseases [120].

Elevated levels of IL-1 prior to astrogliosis were detected, which suggest the involvement of IL-1 in the regulation of astrocyte reactivity associated with increased GFAP and S100B levels [121,122]. The complex interactions between APP, astrocytes activation, S100B, and IL-1 include upregulation of the expression of IL-1α and -1β by both APP and S100B [123,124], and induction of both APP and S100B by IL-1β. Such interactions stimulate multiple neural insults, gliosis-related neuroinflammation and risk for development of the neuropathological changes associated with DS [123,124,125]. Cytokine overexpression and astrocyte activation occurs years before the appearance of Aβ plaques at middle age in DS [123,124,126]. This observation supports the cytokine hypothesis which was proposed previously and indicates that risk factors, genetic and environmental, activate glial cells and produce excess amounts of IL-1 and S100B before Aβ-related pathology [127], which suggest a role for astrocytes in plaque formation.

### 2.4. Frontotemporal Dementia

Frontotemporal dementia (FTD) is a heterogeneous neurodegenerative disease with different aspects of language and behavioral deficiency with asymmetrical focal atrophy of the temporal and/or frontal lobes on the brain. FTD has been classified clinically into three subtypes: semantic dementia (SD), behavioral variant of FTD (bvFTD), and progressive nonfluent aphasia with the bvFTD as the most common type [128]. Clinically, FTD is characterized by non-cognitive symptoms such as change in eating habits, repetitive behaviors, hyperorality and by early changes in personality including: euphoria, disinhibition, and apathy. In addition, it is represented with progressive impairment of executive functions and language [129]. Behavioral variant FTD is presented with changing in interpersonal, emotional, and social behaviors [130], but in primary progressive aphasia, nonfluent verbal output remains the only symptoms for at least two years [129]. Frontotemporal dementia usually starts earlier than other types of dementia such as AD [131] and it is the most frequent presenile dementia in those less than 65 years of age [132]. The prevalence of FTD is 15 per 100,000 in subjects between 45 and 65 years old with mean survival rate from three to 10 years from diagnosis [133]; and it is known that 40% of FTD cases are familial [134], and 10–30% of the familial FTD patients have a mutation in the microtubule-associated protein tau (MAPT) gene [135].

Other neurodegenerative diseases such as AD may present with frontal lobe symptoms, therefore differential diagnosis may be difficult. Different biological markers such as MAPT and Aβ_42_ can be used to differentiate between AD and FTD [129]. There is an increased production of Aβ_42_ in patients with AD, which leads to the formation of senile plaques located extracellularly. The increased brain deposition of Aβ_42_ in addition to the diminished number of neurons, which produce Aβ_42_, results in lower concentration of Aβ_42_ in the CSF of AD patients compared to the control subjects and to patients with other dementias [31,32]. The concentrations of Aβ_42_ in patients with FTD are between those of control and AD patients. The Aβ_42_ concentrations are lower in the CSF of FTD patients than in control subjects [129,136], but much higher than that in AD patients [137,138,139]. However, there is no significant difference in Aβ_42_ concentrations between the three subtypes of FTD [129]. Increased levels of CSF Aβ_42_ in FTD patients were not related to the cognitive performance or the duration of the disease [129]. The lack of such association suggested the neurotoxicity mediated by Aβ_42_ may not have a role in FTD [129]. In contrast, the level of Aβ_40_ in the CSF was found to be normal in AD [140]; while in frontotemporal lobar degeneration (FTLD) the level of Aβ_40_ was significantly lower when compared to controls and AD patients [141]. Accordingly, the ratio of CSF Aβ_42_ to Aβ_40_ could be used to differentiate between AD and FTLD where this ratio decreases in AD but not in FTLD patients when compared to control subjects. The low level of Aβ_40_ in FTLD patients was confirmed by other studies [142,143,144,145]. In a post-mortem study [146], the authors reported the presence of Aβ_42_ containing plaques in FTLD patients with occasional Aβ_40_ plaques. Other studies, however, which monitored CSF-Aβ_42_ levels in FTLD patients and reported higher CSF Aβ_42_ compared to AD subjects argued against Aβ plaques formation due to elevated Aβ_42_ levels in the CSF [129,138]. Yet, a strong association between CSF Aβ_42_ and cognition was estimated in patients with bvFTD where low CSF Aβ_42_ concentration was associated with worse executive function and with worse general cognitive dysfunction, suggesting the pathological role of Aβ_42_ in bvFTD [147].

Frontotemporal dementia may have three histopathological changes [148]. One type has microvacuolation of the anterior and frontal temporal lobes with an increase in the number of astrocytes in the outer layer of the cortex. Another type has gliosis in the cortical layers with tau inclusion and ballooned neurons [148]. The third histopathological change includes either of the above changes with loss of motor neurons in the anterior horn of the spinal cord [148]. A previous study reported that the majority of FTD patients exhibit a significant number of reactive astrocytes in the outer layer of the temporal and frontal cortex as compared to control [149], which was confirmed by the increased expression of GFAP [150]. This finding may reflect significant reactive astrocytosis in FTD patients. In addition to astrogliosis, gliosis in the temporal and frontal cortices was observed. The significantly high levels of gliosis observed in the temporal and frontal cortices compared to the occipital and parietal regions may indicate that FTD starts first in the temporal and the frontal regions [149,151], which may progress to the occipital and parietal regions in the late stages of FTD [149]. Astrogliosis occurs in areas with less neuronal loss, *i.e.*, in the occipital and parietal cortices, which then advances to other areas with neuronal damage as FTD progresses [149]. Broe and colleagues reported that the degree of frontotemporal atrophy is directly related to the degree of astrocytes apoptosis and loss of astrocytic support in FTD subjects, and is independent from histopathological mechanisms but related to the disease duration and its clinical severity [152,153]. Furthermore, astrocyte apoptosis, which is correlated with the degree of neuronal atrophy and loss, occurs in the earliest disease stages in FTD when there is mild frontotemporal atrophy [152]. This suggests that progressive neuronal loss in FTD may be due to other cell death mechanisms [152]. On the other hand, previous analysis of FTD revealed classical features of apoptosis in both astrocytes and neurons [149,154,155]. At the late disease stages, GFAP-positive astrocytes increased due to loss of tissues and neurons at these stages [152]. However, the neuronal loss in FTD was disputed by others, who reported neuronal apoptosis to be minimal even in the early stages of FTD [156]. Kersaitis *et al.* [157] examined cases of sporadic bvFTD and revealed severe astrogliosis in both temporal and frontal cortices and neuronal loss in the upper cortical layers of the frontal lobe. This may indicate that there is an association between FTD stages and both astrocyte upregulation and frontal lobe neuronal loss [158]. In addition, it may indicate that astrocytosis helps in the initial preservation of cortical tissue [157].

Studies which correlate the role of astrocytes in FTD with Aβ level are lacking, therefore, the association between astrocytes and Aβ in FTD patients may have the same correlation as in AD [38,47,56,65]. Further studies, however, are required to determine this association in FTD.

## 3. Conclusions

In this review, we presented the interplay between Aβ and astrocytes and their contribution to neuroinflammation and neurotoxicity observed in Aβ-related pathologies including AD, CAA, DS and FTD. Neuroinflammation is currently recognized as a central pathophysiological feature of these neurodegenerative disorders. This suggests similarities between these disorders and that the development of a treatment for one disorder could be beneficial in the treatment of the other. At the time when patients have signs and symptoms, Aβ pathology would have already occurred [159]. Thus, early pretreatment with novel therapies that induce Aβ clearance and reduce brain inflammatory burden is expected to reduce the risk of Aβ related pathologies. Current AD treatments, which have also been used or tested in patients with CAA, DS and FTD [160,161,162,163], focuses on the inhibition of acetylcholinesterase enzyme (the ChEI drugs donepezil, rivastigmine and galantamine) and blocking the glutamate receptor NMDA (memantine). Available reports support alternative mechanisms by which these drugs work beside their original mechanisms of action. Preclinical studies in AD transgenic mice demonstrated the ChEI drugs and memantine to alter Aβ deposition in the brains of transgenic animals [164,165,166,167,168]. For example, galantamine treatment was able to reduce Aβ load and improve Aβ-induced cognitive dysfunction in the AD model 5xFAD [164], and to decrease formation of toxic Aβ oligomers [165]. Similar effects were also seen with donepezil and rivastigmine where both drugs increased the clearance of exogenously administered Aβ in wild type animals [166]. Further studies with rivastigmine in an AD mouse model demonstrated induced clearance of brain Aβ by upregulating P-gp and LRP1 proteins, which was associated with reduced astrogliosis and inflammatory markers [167]. Additionally, memantine reversed cognitive impairment in AD transgenic mice indicating the importance of glutamate toxicity in Aβ pathology [168]. We thus support the notion that the clinical efficacy of available AD drugs is due not only to activation of the cholinergic pathway or inhibiting NMDA, but also attenuation of Aβ-mediated neurotoxicity. Despite this effect of AD drugs on Aβ and their activation of cholinergic transmission (ChEIs) or NMDA inhibition (memantine), these agents are not capable of decreasing or altering the progression of AD. Donepezil, for example, when investigated in patients with mild to moderate AD in a placebo-controlled double-blind trial for its effect on the cognitive function, caused no difference between the treatment and placebo groups [169], which might indicate that ChEIs do not alter the course of AD. However, in most of the cases, ChEIs are prescribed to patients who already developed AD or at late stages of the disease, where irreversible neuronal damage and potential Aβ deposition and plaques are evident, and therapeutic benefits are expected to be very limited. This same reason could also explain the multiple failures reported with other tested therapeutics like anti-inflammatory drugs and immunotherapy in clinical trials. Thus, administering these agents to patients at very early stages of AD or patients who are at high risk of developing the disease is proposed, which necessitate early diagnostic tools for early therapeutic intervention and better curative effect. In addition, since AD drugs’ therapeutic doses are determined based on their cholinergic or NMDA activities, the doses for their non-cholinergic effect may need to be adjusted for better therapeutic efficacy.

All factors associated with Aβ pathology could be thus a legitimate target for a therapeutic intervention, including but not limited to Aβ, astrocytes, cytokines and pro-inflammatory factors. However, due to the complexity of these disorders that are influenced by many factors and exhibit alterations in multiple cellular pathways and processes, in addition to the lack of clarity on whether these factors are a cause or a consequence, it would be beneficial to consider polypharmacology approaches or therapeutics with multiple targets. Examples of such targets are Aβ by increasing its clearance and/or decreasing its production and astrocytes by inhibiting their chronic activation and subsequent neuroinflammatory and oxidative stress effects. Studies from our laboratory in AD and CAA mouse model support interventions with multimodalities that target multiple pathways. Treatment of the AD and CAA mouse model TgSwDI with oleocanthal, a phenolic secoiridoid component of extra-virgin olive oil (EVOO) with potent anti-inflammatory effect comparable to that of the drug ibuprofen [170], demonstrated a protective and therapeutic potential against AD and CAA. Mice treatment with oleocanthal caused a significant reduction in amyloid load in the brain and microvesselsas by multiple mechanisms, a) enhanced brain clearance of Aβ across the BBB by increasing the expression of major amyloid clearance proteins at the BBB including P-gp and LRP1, and b) activated the ApoE-dependent amyloid clearance pathway in mouse brain. This reduction was in addition to oleocanthal anti-inflammatory effect where it was able to reduce astrocytes activation and IL-1β levels [63].

While most research and clinical studies are directing their efforts toward the development of therapeutics that target Aβ, there are sparse number of studies reported which tested the potential of astrocyte-targeted therapeutics [171,172,173,174]. In these studies, multiple mechanisms were investigated including S100B inhibition by pentamidine [171], reduction of proinflammatory responses of microglia and astrocytes via the inhibition of monoacylglycerol lipase by JZL184 [172], enhancement of the astrocytes’ lysosomal function by activating the transcription factor EB [173], and alteration in the immune/inflammatory signaling pathway by administering adeno-associated virus vectors containing the astrocyte-specific Gfa2 promoter [174]. The promising findings from these studies support the deleterious effect of activated astrocytes in AD and other related diseases, and highlight the need to explore novel astrocyte-based therapies.

In conclusion, astrocytes play important roles in the regulation of brain homeostasis, protection and function by supporting neuronal health and activity; however astrocytes also contribute largely to neuronal degeneration in pathological situations that could further be emphasized by Aβ. Thus, there is a serious need for developing novel strategies that target Aβ and astrocytes as both are recognized to play a key role in the etiology of Aβ-related disorders.

## Abbreviations

ACE	Angiotensin-Converting Enzyme
AD	Alzheimer’s Disease
ADDLs	Amyloid Derived Diffused Ligands
APP	Amyloid Precursor Protein
Aβ	Amyloid Β
BACE-1	Secretase-b
BBB	Blood-Brain Barrier
bvFTD	Behavioral Variant Frontotemporal Dementia
CAA	Cerebral Amyloid Angiopathy
CBF	Cerebral Blood Flow
CNS	Central Nervous System
COX-2	Cyclooxygenase-2
CSF	Cerebral Spinal Fluid
DS	Down Syndrome
FTD	Frontotemporal Dementia
FTLD	Frontotemporal Lobar Degeneration
GFAP	Glial Fibrillary Acidic protein
GLT-1	Glutamate Transporter
GLUT1	Glucose Transporter
ICH	Intracerebral Hemorhage
IDE	Insulin-Degrading Enzyme
IL-1	Interleukin-1
IL-1α	Interleukin-1α
IL-1β	Interleukin-1β
LRP1	Lipoprotein Receptor Related Protein 1
MAPT	Microtubule-Associated Protein Tau
MMPs	Matrix Metalloproteinases
NADPH	Nicotinamide Adenine Dinucleotide Phosphate
NEP	Neprilysin
NF-κB	Nuclear Factor-Kappa B
NMDA	*N*-methyl-d-aspartate
Pgp	P-glycoprotein
RAGE	Receptor for Advanced Glycation End Products
ROS	Reactive Oxygen Species
SD	Semantic Dementia
TNF-α	Tumor Necrosis Factor-α
TEER	Transepithelial Electrical Resistance
